# Patient experience survey for community drug and alcohol service users in hospitals

**DOI:** 10.1192/bjo.2021.611

**Published:** 2021-06-18

**Authors:** Nurul Yahya, Derrett Watts

**Affiliations:** North Staffordshire Combined Healthcare NHS Trust

## Abstract

**Aims:**

To explore and monitor experience of hospital care provided to patients of Stoke Community Drug and Alcohol Services (CDAS) and Edward Myers Unit (EMU; detox inpatient based unit).

**Method:**

The sample was collected from patients who attended face-face clinics at CDAS and patients living in Stoke-On-Trent who were admitted to the Edward Myers Unit. The survey pertains to four locations, which include Royal Stoke Hospital, A + E, Harplands Hospital (Mental Health Unit), and EMU.

We collected data of over two months from September–November 2020. The cohort of patients from CDAS included new presentations or restart Opioid Substitution Treatment (OST) clinics and people known to the alcohol team at CDAS.

We delivered a survey pertaining to experience of hospital care in the last 12 months. This includes treatment at A&E Royal Stoke Hopital, any of the wards at Royal Stoke Hospital, Harplands Hospital and Edward Myers Unit.

**Result:**

The uptake for the survey was 53/83 (64%) at CDAS clinic and 23/44 (52%) at Edward Myers Unit. The sample comprised more men than women. The majority were aged 31–40 years. Most common substances used were alcohol.

Majority of patients has been admitted to the general hospital, either in the ward or seen at A + E. Most people were very satisfied with their treatment in all four locations. This include withdrawal symptoms, pain, mental health, and discharge plan. There were diverse reasons given of the satisfactory scores. EMU seems to have the best overall scores comparatively to the other units, with Harplands Hospital seems to be doing worse.

The free text comments revealed that the staffs' courtesy, respect, careful listening and easy access of care was particularly the strongest driver of overall patient satisfaction. Patients look for supportive relationships, to be involved in treatment decisions, effective approaches to care, easy treatment access and a non-judgemental treatment environment. In some aspects, patients were dissatisfied with pain management, longer waiting times and inability to treat them as equal to non drug/alcohol users.

**Conclusion:**

On objective measures, patients were satisfied with treatment received, however, some has point out their dissatisfaction, particularly in the mental health setting. This project calls for greater attention and support for addiction service provision in emergency departments and hospital wards. Although these findings do not represent the views of all patients in SUD treatment, findings give insight into the ways treatment providers, service managers and policy makers might enhance the patient experience to improve patient treatment prognosis and outcomes

